# Effect of Ischemic Preconditioning on the Recovery of Cardiac Autonomic Control From Repeated Sprint Exercise

**DOI:** 10.3389/fphys.2018.01465

**Published:** 2018-10-26

**Authors:** Thiago R. Lopes, Jeann L. Sabino-Carvalho, Thiago H. N. Ferreira, José E. Succi, Antônio C. Silva, Bruno M. Silva

**Affiliations:** ^1^Department of Physiology, Federal University of São Paulo, São Paulo, Brazil; ^2^Laboratory of Exercise Physiology, Olympic Center of Training and Research, São Paulo, Brazil; ^3^São Paulo Association for Medicine Development, São Paulo, Brazil; ^4^Postgraduate Program in Translational Medicine, Federal University of São Paulo, São Paulo, Brazil; ^5^Department of Surgery, Federal University of São Paulo, São Paulo, Brazil

**Keywords:** ischemia, supramaximal exercise, parasympathetic, heart beat, metabolism

## Abstract

Repeated sprint exercise (RSE) acutely impairs post-exercise heart rate (HR) recovery (HRR) and time-domain heart rate variability (i. e., RMSSD), likely in part, due to lactic acidosis-induced reduction of cardiac vagal reactivation. In contrast, ischemic preconditioning (IPC) mediates cardiac vagal activation and augments energy metabolism efficiency during prolonged ischemia followed by reperfusion. Therefore, we investigated whether IPC could improve recovery of cardiac autonomic control from RSE partially via improved energy metabolism responses to RSE. Fifteen men team-sport practitioners (mean ± SD: 25 ± 5 years) were randomly exposed to IPC in the legs (3 × 5 min at 220 mmHg) or control (CT; 3 × 5 min at 20 mmHg) 48 h, 24 h, and 35 min before performing 3 sets of 6 shuttle running sprints (15 + 15 m with 180° change of direction and 20 s of active recovery). Sets 1 and 2 were followed by 180 s and set 3 by 360 s of inactive recovery. Short-term HRR was analyzed after all sets via linear regression of HR decay within the first 30 s of recovery (T30) and delta from peak HR to 60 s of recovery (HRR60s). Long-term HRR was analyzed throughout recovery from set 3 via first-order exponential regression of HR decay. Moreover, RMSSD was calculated using 30-s data segments throughout recovery from set 3. Energy metabolism responses were inferred via peak pulmonary oxygen uptake ($\dot{\text{V}}{\text{O}}_{2}$peak), peak carbon dioxide output ($\dot{\text{V}}{\text{O}}_{2}$peak), peak respiratory exchange ratio (RERpeak), first-order exponential regression of $\dot{\text{V}}{\text{O}}_{2}$ decay within 360 s of recovery and blood lactate concentration ([Lac-]). IPC did not change T30, but increased HRR60s after all sets (condition main effect: *P* = 0.03; partial eta square (η^2^_*p*_) = 0.27, i.e., large effect size). IPC did not change long-term HRR and RMSSD throughout recovery, nor did IPC change any energy metabolism parameter. In conclusion, IPC accelerated to some extent the short-term recovery, but did not change the long-term recovery of cardiac autonomic control from RSE, and such accelerator effect was not accompanied by any IPC effect on surrogates of energy metabolism responses to RSE.

## Introduction

Training with repeated sprint exercise (RSE) requires less time per session (Stork et al., 2017) and induces similar or even superior feelings of satisfaction (Stork et al., 2017) and aerobic adaptations (Gist et al., 2014) compared to continuous endurance training. Sprint-based training has therefore been considered as a valuable strategy to improve health-related physical fitness of subjects with little time available to engage in regular programs of exercise training (Gist et al., 2014; Stork et al., 2017). However, heart rate (HR) recovery (HRR) is acutely slower and recovery of time domain heart rate variability (HRV) is acutely blunted post-RSE as compared with post-moderate continuous exercise matched to net energy expenditure (Buchheit et al., 2007; Nakamura et al., 2009; Del Rosso et al., 2017). Of note, the acute slowing of HRR after RSE may raise clinical concerns, because slow HRR from maximal exercise, particularly within the first 60 s of recovery (i.e., short-term recovery), is strongly associated with increased risk of cardiac events in subjects with cardiovascular risk factors (Cole et al., 1999).

Post-exercise HRR and HRV are assumed to be mostly determined by interplay between cardiac vagal reactivation and sympathetic withdrawal to the sinus node (Goldberger et al., 2006; Coote, 2010; Peçanha et al., 2014). Thus, methods that improve the cardiac autonomic control could possibly attenuate the acute effect of RSE on post-exercise HRR and HRV. In this context, non-lethal brief cycles of ischemia-reperfusion [i.e., ischemic preconditioning (IPC)] at a site (e.g., limb) induce powerful protection against injury provoked by prolonged ischemia and subsequent reperfusion at a remote site (e.g., heart) (Kharbanda et al., 2002), and the vagal branch of the autonomic nervous system seems to play a pivotal role in such IPC-mediated protection. The reason is that, in rats, vagotomy (Basalay et al., 2012), blockade of muscarinic receptors with atropine (Mastitskaya et al., 2012) or optogenetic silencing of vagal pre-ganglionic neurons (Mastitskaya et al., 2012) nullified the IPC protection against ischemia-reperfusion injury. In addition, IPC not only protects against injury, but may improve some healthy phenotypes (Cocking et al., 2018; Jeffries et al., 2018). For instance, IPC increased resting high-frequency HRV (i.e., a surrogate of cardiac vagal control) in healthy uninjured men (Enko et al., 2011). Therefore, evidence from both ischemia-reperfusion models and healthy resting humans supports that IPC exerts an excitatory effect on the vagal branch of the autonomic nervous system. However, it remains untested whether IPC accelerates post-exercise HRR and augments post-exercise time domain HRV, particularly during the short-term recovery period, in which HRR and time domain HRV are mostly determined by the vagal control of the heart. Testing this issue in healthy humans could then motivate further studies in subjects at risk for cardiovascular events, in case the IPC shows a promising beneficial effect.

If IPC improves post-exercise HRR and time-domain HRV, the IPC effect could be mediated by two non-excluding possibilities. On the one hand, mechanisms that mediate the IPC vagal excitation in models of ischemia-reperfusion injury could play a role (Gourine and Gourine, 2014). These mechanisms include activation of afferent pain fibers at the preconditioned site, as well as release of substances in the circulation from the preconditioned site (Gourine and Gourine, 2014). Thus, one plausible hypothesis is that neural and humoral mechanisms triggered at the preconditioned site could directly activate cardiac vagal neurons leading to a possible beneficial effect of IPC on post-exercise HRR and time domain HRV. On the other hand, IPC could improve post-exercise HRR and time domain HRV via reduction of the exercise-induced energy metabolism distress, which could indirectly increase the cardiac vagal control. Two sets of evidence support such energy metabolism hypothesis. Firstly, manipulation of the aerobic and anaerobic lactic energy contribution to the total energy expenditure of exercise showed that the lower the contribution of the anaerobic lactic metabolism, the faster is the HRR (Buchheit et al., 2007; Nakamura et al., 2009; Del Rosso et al., 2017). The underlying reason for this phenomenon is unknown, but it may involve less metabolites buildup leading to less activation of metabolite-sensitive receptors like muscle metaboreceptors and carotid chemoreceptors (Buchheit et al., 2007). Secondly, IPC augments energy metabolism efficiency in skeletal muscles of pigs exposed to prolonged ischemia (Pang et al., 1995), presumably due to increased efficiency of the mitochondrial electron transport chain (Garlid et al., 2003; Thaveau et al., 2007; Cabrera et al., 2012). Furthermore, some studies have reported IPC to increase peak pulmonary oxygen uptake ($\dot{\text{V}}{\text{O}}_{2}$) (de Groot et al., 2010; Cruz et al., 2015) and decrease blood lactate concentration [Lac-] (Bailey et al., 2012) during incremental dynamic exercise in moderately trained subjects, suggesting increased aerobic and decreased anaerobic lactic contribution to exercise energy metabolism. Others have reported IPC not to modify energy metabolism responses to RSE (Patterson et al., 2015; Griffin et al., 2018), but perhaps the IPC dose (1-day exposure) that was effective for incremental dynamic exercise may not be sufficient for RSE. Herein, we chose to test the later hypothesis (i.e., energy metabolism hypothesis) and we employed an IPC dose (3-day exposure) greater than previous studies that investigated the IPC effect on energy metabolism responses to RSE (Patterson et al., 2015; Griffin et al., 2018). Thus, we investigated whether repeated exposure to IPC could improve recovery of cardiac autonomic control from RSE partially via improved energy metabolism responses to RSE.

## Methods

### Subjects

Fifteen healthy men participated in the study (mean ± standard deviation: 25 ± 5 years, 81.2 ± 9.7 kg and 179.3 ± 7.4 cm). Subjects were engaged in some type of physical training at least three times a week during the last year and in non-professional team sport competitions (e.g., soccer and basketball). All subjects provided written informed consent before participating in the study. The study conformed to the Declaration of Helsinki and was approved by the Ethics Committee of the Federal University of São Paulo (process number: 192.224).

### Experimental design

**Figure 1:**
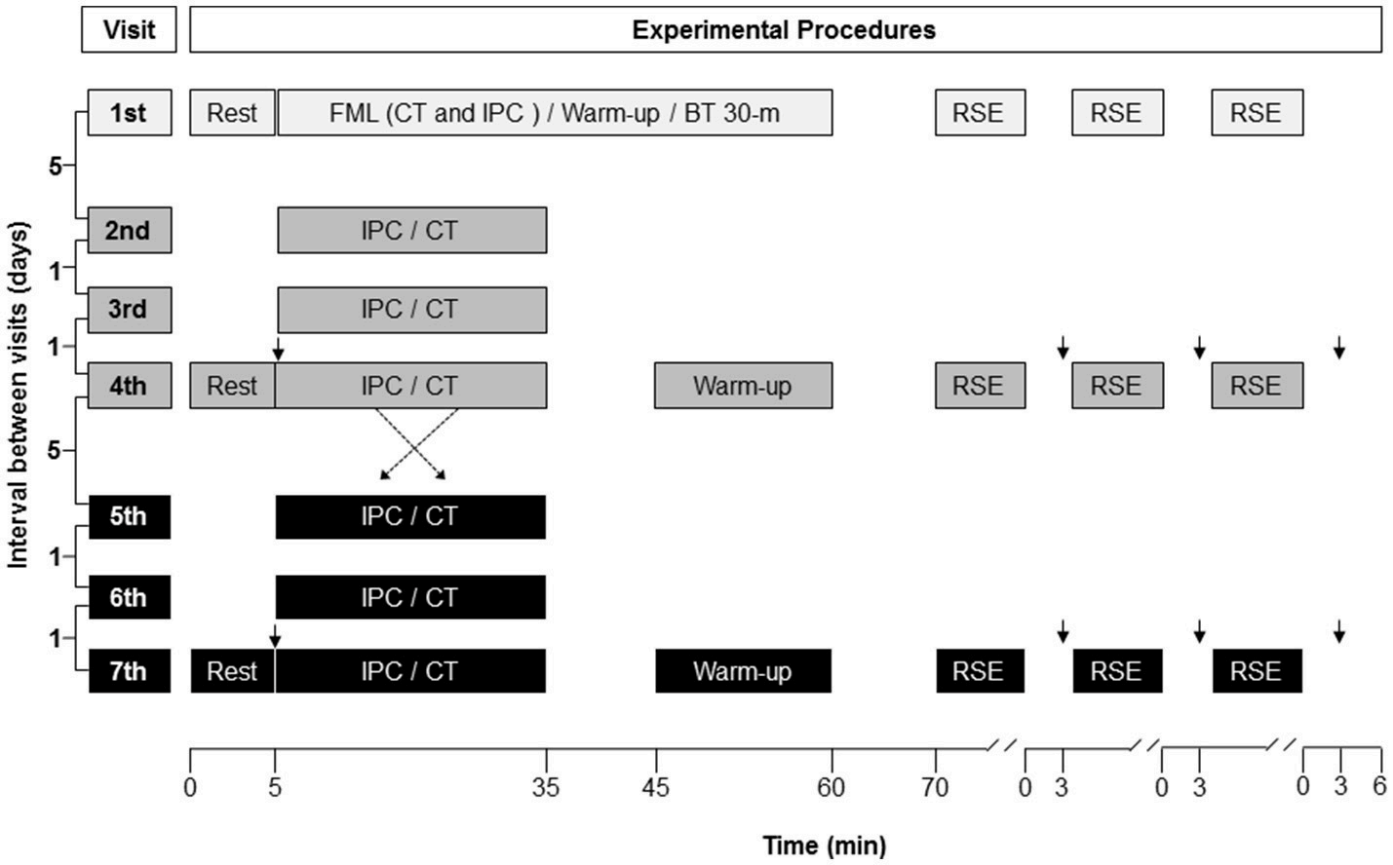
Illustration of the experimental design. The protocol was randomized, controlled, crossed-over and double-blinded. FML, familiarization; CT, control procedure; IPC, ischemic preconditioning; BT 30-m, best time in a 30-m sprint, with a change of direction of 180° at the middle of the course (15 + 15 m); RSE, repeated sprint exercise; ↓, blood sample for blood lactate analysis.

The study was single-blinded, crossed-over, randomized and controlled. Each subject visited the laboratory on seven occasions (Figure 1). Just one subject was assessed at a time. The first visit was used for familiarization and measurement of the best time in a 30-m sprint, with a change of direction of 180° at the middle of the course (i.e., 15 + 15 m). Subjects had three to six trials, separated by 180 s of inactive recovery, to achieve their best time (BT). Then, the BT served as reference in the RSE task to check an all-out pacing strategy. Exposure to IPC and CT occurred on three occasions, 48 and 24 h before the RSE task, as well as on the day of the RSE task. The last exposure to IPC and CT finished 35 min before the onset of the RSE task. Likewise previous studies (de Groot et al., 2010; Crisafulli et al., 2011; Bailey et al., 2012; Barbosa et al., 2015; Cruz et al., 2015; Del Rosso et al., 2017), subjects were not informed about the study hypothesis in an attempt of blinding. RSE was performed in a sports court, at the same time of the day, with interval between each test of at least 7 and most 14 days. Subjects were instructed to eat a light meal 2 h before RSE, as well as not to train and consume caffeine and alcohol 24 h before RSE.

### Ischemic preconditioning and control

IPC was performed with subjects seated using customized cuffs which were specifically made to occlude the circulation at the tights (Ferreira et al., 2016). One cuff was used per tight. The cuff had two independent bladders mounted in series. Each bladder was 36 cm long and 17.5 cm wide. Together the bladders covered at least 80% of a thigh's circumference. An aneroid manometer and an inflation bulb were attached to each bladder. Each cuff had a Velcro strap which, in most cases, surrounded a tight at least two times. In the IPC procedure, cuffs were inflated one at a time to 220 mmHg for 5 min. A total of three inflation/deflation cycles were performed per leg (de Groot et al., 2010). Cuff inflation generally took 15–25 s. Occlusion time started to be counted after the target pressure was achieved. In the CT procedure, cuffs were inflated to 10 mmHg for 5 min and deflated to 0 mmHg for 5 min.

The target IPC inflation pressure of 220 mmHg should be more than enough to cause arterial and venous occlusion in the tights of normotensive subjects using large cuffs as ours. Even so, we used a vascular Doppler (Doppler vascular 610B, MEDMEGA, Brazil) to guarantee the presence of arterial occlusion. The site that yielded the best blood flow signal at the posterior tibial artery was identified at the beginning of each experimental day. The skin was marked at this site. The head of the Doppler transducer was positioned at the marked site at pre-inflation and maintained at this place while a cuff was inflated. The signal usually disappeared when the cuff pressure achieved 140–160 mmHg. Thus, at the target pressure no flow signal was present in any inflation in any subject.

IPC yields two windows of protection against ischemia-reperfusion injury (Hausenloy and Yellon, 2010). The first begins immediately after the IPC exposure and lasts about 2 h (Hausenloy and Yellon, 2010). The second begins approximately 12–24 h after the IPC exposure and can last for 48–72 h (Hausenloy and Yellon, 2010). Of note, repeated exposure to IPC before the second window effect is over has been shown to amplify the IPC effect on the resting vascular function in humans (Loukogeorgakis et al., 2005; Jones et al., 2014). Although an effective dose and timing of IPC administration has not yet been determined for the sake of improvement of exercise-related responses, evidence indicates that the IPC ergogenic effect may last for many hours (Lisbôa et al., 2017). Hence, in the present study, IPC was applied on two consecutive days before, as well as on the day of the RSE task in an attempt to sum early and late effects of IPC (Loukogeorgakis et al., 2005), which could amplify the IPC effect (Jones et al., 2014). However, the use of this design precluded identification of the effective dose of IPC (i.e., the effects from just the acute or repeated exposure could be responsible).

### Repeated sprint exercise

The warm-up began 10 min after IPC or CT exposure. The warm-up consisted of a standardized and supervised exercise routine that lasted 15 min, including moderate intensity running (5 min), athletic drills (Anfersen and Skipping), dynamic stretching and four maximal accelerations (15 m) with a change of direction at the end (180°), separated by 1 min of active recovery (walking). Once the warm-up ended, a portable metabolic analyzer (K4b2, Cosmed, Italy) was placed in the subjects, which usually took approximately 10 min.

The RSE task consisted of three sets (i.e., bouts) of six sprints. Each sprint was 30-m long, with a change of direction of 180° at the middle of the course (15 + 15 m). There were 20 s of active recovery between sprints, 180 s of inactive recovery between sets and 360 s of inactive recovery after the last set. During the active recovery, subjects slowed down (10 m), scrolled through a 24-m course during 15–17 s, and then waited for 3–5 s at the start position for the sound signal of the next sprint (Supplementary Video 1). Sound signals were automatically emitted by a photocell system (Test Speed 6.0, CEFISE, Brazil). Pace at the recovery course was verbally informed in order to maintain a correct scroll rhythm. At the end of each set, subjects rapidly slowed down and sat on a chair positioned beside the recovery course (10 m after the finish line).

Subjects were instructed to run as fast as possible during every sprint and were verbally encouraged throughout the test. The researcher that gave verbal encouragement was blinded to the IPC or CT exposure. Subjects had to achieve in the first sprint of the first set at least 95% of their BT obtained in the familiarization visit. Importantly, RSE has been shown to be reproducible and valid for assessing repeated sprint ability in team sports athletes (Rampinini et al., 2007; Impellizzeri et al., 2008). In addition, the protocol with multiple sets of RSE was chosen because it may resemble more accurately what occurs during team sports games (Serpiello et al., 2011).

### Measurements

HR was recorded beat by beat (S810i, Polar, Finland) and pulmonary gas exchange breath by breath (K4b2, Cosmed, Italy) throughout the RSE task (Hausswirth et al., 1997; Gamelin et al., 2006; Vanderlei et al., 2008; Weippert et al., 2010). However, due to technical problems we did not record long-term HRR data from one subject and $\dot{\text{V}}{\text{O}}_{2}$ data from three subjects. Before each test, O_2_ and CO_2_ analyzers were calibrated according to the manufacturer's specifications using ambient air and gases with known concentration (16% O_2_ and 4% CO_2_). The flowmeter was calibrated using a 3-L syringe. An ointment was used in an ear lobe to induce vasodilation at pre-interventions and pre-RSE (Finalgon, Boehringer Mannheim, Germany). Then, a capillary blood sample (25 μL) was collected from the earlobe in a heparinized and calibrated capillary before the exposure to IPC and CT, as well as at 180 s of recovery after each RSE set. Each blood sample was stored in an Eppendorf containing 50 μL of 1% NaF (i.e., anticoagulant) and frozen at −20°C until analysis of blood lactate concentration ([Lac-]) (YSI 1500 SPORT, Yellow Springs Instruments, USA). Time of each sprint was measured with an accuracy of 0.001 s via the photocell system (Test Speed 6.0, CEFISE, Brazil). A photocell was placed 50 cm above the ground. Subjects had to stay 30 cm behind the photocell to avoid a false start at sprint departure.

### Data analysis

R-R intervals were extracted from the heart rate monitor and placed in a customized Excel spreadsheet. The difference between consecutive R-R intervals varied from 0 to 20% for 99% of all R-R intervals recorded in the study (29,636 R-R intervals). As we assessed young healthy mean, abnormal data most probably represented measurement artifacts due to bad contact between the thorax strip and the underling skin, rather than extra-systoles. Abnormal data were objectively identified by an automatic filter that highlighted any R-R interval differing more than 20% from the previous one. This procedure should preserve the physiological variability between successive R-R intervals, while removing artifacts and unlikely extra-systoles (Task Force, 1996). Abnormal data were then deleted and replaced by linear interpolation of adjacent data.

HRR from exercise shows a biphasic pattern consisting on a fast HR decay, which lasts about 60 s (Peçanha et al., 2014), followed by a slow HR decay, which is usually analyzed up to 360 s of recovery (Peçanha et al., 2014). Therefore, HR data recorded during the first 60 s of recovery from sets 1, 2 and 3 were used to calculate the following short-term indexes of the cardiac autonomic control (Buchheit et al., 2007; Nakamura et al., 2009; Peçanha et al., 2014): (1) negative reciprocal of the slope obtained from a linear regression between natural log-transformed HR and time using data from the first 30 s of recovery (T30); (2) absolute difference between 5-s mean HR at the end of a set (HRpeak) and 60 s later (HRR60s). HR data recorded until 360 s of recovery after set 3 were used to calculate a time constant (Tau) of a first-order exponential decay regression. HRR Tau therefore represented a long-term index of the cardiac autonomic control (Buchheit et al., 2007; Nakamura et al., 2009; Peçanha et al., 2014). In addition, RMSSD of 30 s data segments (i.e., RMSSD) provided an index of HRV from the onset to the end of set 3 recovery (Buchheit et al., 2007; Nakamura et al., 2009; Peçanha et al., 2014). T30, HRR60s and RMSSD were calculated in a customized Excel spreadsheet. Tau was calculated in the Origin 6.0 software (Microcal, USA). T30, HRR60s, Tau and RMSSD have shown to be valid indexes of cardiac autonomic control at post-exercise via pharmacological blockade studies (Imai et al., 1994; Goldberger et al., 2006). The reported coefficient of variation for these indexes after high-intensity intermittent exercise is: T30 = 73% (Dupuy et al., 2012), HRR60s = 11% (Bonato et al., 2018), Tau = 14% (Bonato et al., 2018) and RMSSD = 15–28% (Al Haddad et al., 2011). The investigator that analyzed all the data (T.R.L.) was not blinded to the conditions. However, raw R-R intervals were objectively processed. Then, data were used for calculations using fixed mathematical parameters. Therefore, no step in the analysis process of R-R intervals was vulnerable to subjective data handling, and the same applies to the breath data analysis described next.

Breathing data were filtered to exclude aberrant breaths (two standard deviations from the mean of a 30-breath window) (Poole and Jones, 2012). Valid breath by breath values were linearly interpolated to get one value per second (Origin 6.0, Microcal, USA). Then, 5-s means were calculated. Peak oxygen uptake ($\dot{\text{V}}{\text{O}}_{2}$peak) and carbon dioxide output ($\dot{\text{V}}{\text{O}}_{2}$peak) were taken as: (1) the highest 5-s mean of the last sprint of each set, and (2) the mean of the three highest 5-s values of each set. The two analyses provided similar results' interpretation, and so only the results from the former were presented. Peak respiratory exchange ratio (RERpeak) was calculated dividing $\dot{\text{V}}{\text{O}}_{2}$peak by $\dot{\text{V}}{\text{O}}_{2}$peak. Kinetics of long-term $\dot{\text{V}}{\text{O}}_{2}$ recovery was represented by the Tau of a first-order exponential decay regression using $\dot{\text{V}}{\text{O}}_{2}$ data from the end of set 3 to the end of the subsequent 360-s recovery period (Rossiter et al., 2002). $\dot{\text{V}}{\text{O}}_{2}$ recovery kinetics was measured because of its association with phosphocreatine recovery kinetics (Rossiter et al., 2002), which, in turn, is largely dependent on the oxidative metabolism (Piiper and Spiller, 1970). Accumulation of blood [Lac-] (Δ[Lac-]) was quantified via deltas of two consecutive measurements (i.e., set 1 minus baseline; set 2 minus set 1; and set 3 minus set 2). At last, the following parameters were obtained to assess RSE performance: BT, total time (TT) and percent sprint performance decrement (%DC). The %DC was determined as follows: (100^*^(TT/ BT^*^6))-100 (Fitzsimons et al., 1993). The reported coefficient of variation for the BT, TT and %DC is 1.3, 0.8, and 30.2%, respectively (Impellizzeri et al., 2008).

### Statistical analysis

Sample size was calculated taking into account a two-way repeated measures ANOVA with two interventions (IPC and CT) and three repeated measures (sets 1, 2, and 3). The main endpoint was the HRR60s, given that this is valid (Kannankeril et al., 2004), reproducible (Al Haddad et al., 2011; Dupuy et al., 2012; Bonato et al., 2018) and widely used in clinical and sports settings to assess the post-exercise cardiac autonomic control (Buchheit, 2014; Peçanha et al., 2014). High-intensity interval training protocols provoked significant HRR60s increase (P < 0.05) at *d* effect size of 0.75 (Lamberts et al., 2009) and 0.87 (Villelabeitia-Jaureguizar et al., 2017). Both effects corresponded to an absolute increase of 6 bpm. We reasoned the IPC effect on HRR60s could be approximately half of such effects (d~0.40; absolute delta ~ 3 bpm). Next, the estimated *d* effect size had to be converted to a partial eta square (η^2^_*p*_) effect size to input an estimated effect in a repeated measures ANOVA. This resulted in a η^2^_*p*_ of 0.04. Correlation among repeated measures was at 0.85 and nonsphericity correction at 1.0. Using these parameters, 14 subjects would be necessary to find a *P*-value lower than 0.05, with 0.80 of power (G^*^Power 3.1, Dusseldorf University, Germany).

Data distribution was verified by the Shapiro-Wilk's test. RMSSD did not present normal distribution, and so, was transformed to natural logarithm for inferential analyses. Paired Student's *t*-test was used to analyze HRR Tau, baseline [Lac-] and $\dot{\text{V}}{\text{O}}_{2}$ Tau. Two-way repeated measures ANOVA (factors: condition and set) was used to analyze HRpeak, short-term HRR, $\dot{\text{V}}{\text{O}}_{2}$peak, $\dot{\text{V}}{\text{O}}_{2}$peak, RERpeak, [Lac-], Δ[Lac-], and RSE performance. Two-way repeated measures ANOVA (factors: condition and time) was used to analyze 5-s mean HR and RMSSD along 360 s after set 3. Three-way repeated measures ANOVA (factors: condition, set and time) was used to analyze 5-s mean HR throughout 60-s recovery periods after all sets. The Greenhouse-Geisser's correction was used to adjust ANOVA results, whenever sphericity was violated in the Mauchly's test. The LSD *post hoc* was used when significant F values were found. Effect sizes for Student's *t*-test and ANOVA results were calculated as Cohen's *d* and η^2^_*p*_, respectively. The following thresholds were used for *d* and η^2^_*p*_ interpretation (Cohen, 1988): *d*, trivial > 0.00; small > 0.20; medium > 0.50; and large > 0.8/η^2^_*p*_, trivial > 0.00; small > 0.01; medium > 0.06; large > 0.14. Results are presented as mean ± standard error of mean (SEM). Statistical significance was set at *P* < 0.05. Statistical analyses were performed in the software Statistica 12 (Statsoft, EUA).

## Results

**Figure 2:**
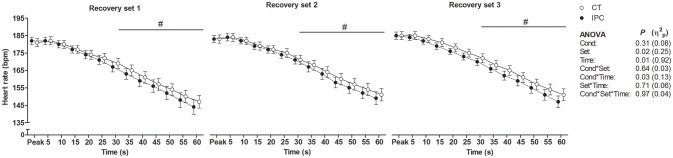
Short-term component of heart rate recovery after each set of repeated sprint exercise. Data were shifted in the X axis to avoid overlapping and thus improve visualization. Data are mean ± SEM. *n* = 15. ^#^*P* < 0.05 between conditions in the *post hoc* analysis of Cond ^*^ Time interaction. CT, control; IPC, ischemic preconditioning; Cond, condition; η^2^_*p*_, partial eta square.

**Figure 3:**
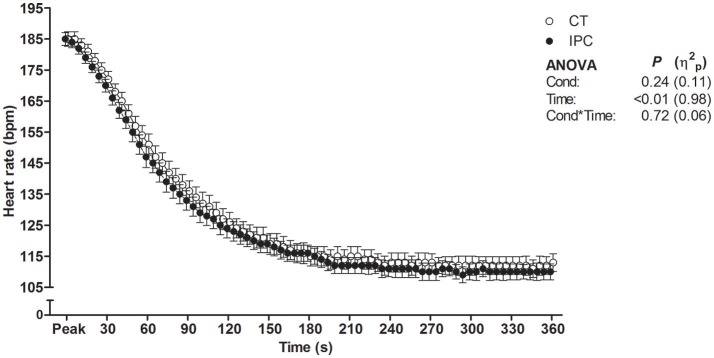
Heart rate throughout 360 s of recovery after set 3. Data were shifted in the X axis to avoid overlapping and thus improve visualization. Data are mean ± SEM. *n* = 14. CT, control; IPC, ischemic preconditioning; Cond, condition; η^2^_*p*_, partial eta square.

**Figure 4:**
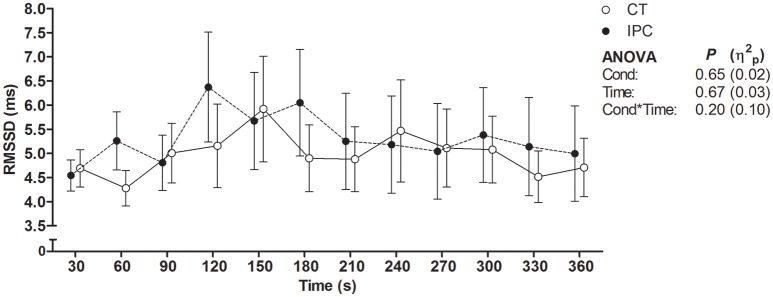
Root mean square of successive differences between R-R intervals (RMSSD) throughout 360 s of recovery after set 3. Data were shifted in the X axis to avoid overlapping and thus improve visualization. Data are mean ± SEM. *n* = 14. CT, control; IPC, ischemic preconditioning; Cond, condition; η^2^_*p*_, partial eta square.

HR from peak to 25 s of recovery was similar between IPC and CT in all sets (Table 1 and Figure 2), leading to similar T30 (Table 1). On the other hand, HR from 30 to 60 s of recovery was lower (medium η^2^_*p*_) in the IPC than CT in all sets (Figure 2), and, consequently, IPC increased HRR60s (Table 1; large η^2^_*p*_; change = 12.8%, 95% CI = 5.8–19.7%). HR values were similar between conditions throughout 360 s of recovery from set 3 (Figure 3), resulting in similar HRR Tau (Table 1). RMSSD was also similar between IPC and CT throughout the entire recovery from set 3 (Figure 4). No difference between IPC and CT was observed for baseline [Lac-] (IPC: 1.30 ± 0.16 mmol.L^−1^ and CT: 1.24 ± 0.15 mmol.L^−1^; *P* = 0.45; *d* = 0.20), as well as for $\dot{\text{V}}{\text{O}}_{2}$peak, $\dot{\text{V}}{\text{O}}_{2}$peak, RERpeak, [Lac-] and Δ[Lac-] and $\dot{\text{V}}{\text{O}}_{2}$ Tau (Table 2). Additionally, RSE performance was similar between conditions (Table 3).

**Table 1:** Peak heart rate (HRpeak), short-term and long-term heart rate recovery (HRR) after each set of repeated sprint.

					**ANOVA** ***P*****-value (*****η***^**2**^***_*****p*****_*****)**			**Student's *t*-test *P*-value (*d*)**
		**SET 1**	**SET 2**	**SET 3**	**Condition**	**Set**	**Interaction**	
HRpeak (bpm)	CT	181 ± 2	183 ± 2	185 ± 2	0.76 (0.01)	0.01 (0.33)	0.14 (0.14)	NA
	IPC	182 ± 2	183 ± 2	185 ± 2				
T30 (s)	CT	435 ± 81	434 ± 55	407 ± 48	0.15 (0.16)	0.42 (0.07)	0.48 (0.04)	NA
	IPC	365 ± 56	427 ± 65	374 ± 47				
HRR60s (bpm)	CT	34 ± 3	33 ± 2	34 ± 3	0.03 (0.27)	0.24 (0.09)	0.69 (0.03)	NA
	IPC	39 ± 3	36 ± 3	38 ± 3				
HRR Tau (s)	CT	NA	NA	78 ± 4	NA	NA	NA	0.64 (−0.14)
	IPC			78 ± 6				

*Data are mean ± SEM. T30, negative reciprocal of the slope obtained from a linear regression between natural log-transformed heart rate (HR) and time using data from the first 30 s of recovery; HRR60s, absolute difference between 5-s mean HR at the end of a set and 60 s later; Tau, time constant of a first-order exponential decay regression after set 3; CT, control; IPC, ischemic preconditioning; η${}_{\text{p}}^{\text{2}}$, partial eta square; NA, not applicable; HRpeak, T30 and HRR60s n = 15; HRR Tau n = 14*.

**Table 2:** Energy metabolism responses to repeated sprint exercise.

					**ANOVA** ***P*****-value (*****η***^**2**^***_*****p*****_*****)**		
		**SET 1**	**SET 2**	**SET 3**	**Condition**	**Set**	**Interaction**
$\dot{\text{V}}{\text{O}}_{2}$peak (mL.kg^−1^.min^−1^)	CT	38.45 ± 1.84	37.68 ± 1.64	36.93 ± 1.93	0.95 (0.00)	0.14 (0.17)	0.98 (0.00)
	IPC	38.25 ± 1.74	37.58 ± 1.51	37.02 ± 1.86			
$\dot{\text{V}}{\text{O}}_{2}$peak (mL.kg^−1^.min^−1^)	CT	49.08 ± 1.71	44.32 ± 0.88	42.50 ± 1.42	0.80 (0.01)	<0.01 (0.63)	0.97 (0.00)
	IPC	49.01 ± 1.28	43.96 ± 1.16	42.00 ± 1.66			
RER	CT	1.30 ± 0.05	1.20 ± 0.05	1.18 ± 0.07	0.78 (0.01)	<0.01 (0.63)	0.73 (0.03)
	IPC	1.30 ± 0.05	1.19 ± 0.05	1.15 ± 0.05			
[Lac-] (mmol.L^−1^)	CT	11.05 ± 0.61	14.50 ± 0.96	14.54 ± 1.09	0.81 (0.00)	<0.01 (0.70)	0.84 (0.01)
	IPC	11.06 ± 0.82	14.12 ± 1.11	14.30 ± 1.06			
Δ[Lac-] (mmol.L^−1^)	CT	9.82 ± 0.67	3.44 ± 0.62	0.04 ± 0.41	0.73 (0.01)	<0.01 (0.89)	0.89 (0.01)
	IPC	9.76 ± 0.80	3.06 ± 0.52	0.18 ± 0.61			

*Data are mean ± SEM. $\dot{\text{V}}{\text{O}}_{2}$peak, peak oxygen uptake; $\dot{\text{V}}{\text{O}}_{2}$peak, peak carbon dioxide output; RERpeak, peak respiratory exchange ratio; [Lac-], blood lactate concentration; Δ[Lac-], difference between consecutive blood lactate measures; CT, control; IPC, ischemic preconditioning; η${}_{\text{p}}^{\text{2}}$,partial eta square. $\dot{\text{V}}{\text{O}}_{2}$peak, $\dot{\text{V}}{\text{O}}_{2}$peak and RERpeak n = 12. [Lac-] and Δ[Lac-] n = 15*.

**Table 3:** Repeated sprint exercise performance.

					**ANOVA** ***P*****-value (*****η***^**2**^***_*****p*****_*****)**		
		**SET 1**	**SET 2**	**SET 3**	**Condition**	**Set**	**Interaction**
BT (s)	CT	5.9 ± 0.1	6.0 ± 0.1	6.2 ± 0.1	0.23 (0.10)	<0.01 (0.55)	0.46 (0.05)
	IPC	5.8 ± 0.1	6.0 ± 0.2	6.1 ± 0.1			
TT (s)	CT	37.2 ± 0.4	38.6 ± 0.4	39.4 ± 0.6	0.09 (0.19)	<0.01 (0.70)	0.81 (0.01)
	IPC	36.5 ± 0.3	37.9 ± 0.4	38.6 ± 0.5			
%DC	CT	4.4 ± 0.4	6.7 ± 0.6	6.7 ± 0.8	0.07 (0.21)	<0.01 (0.40)	0.10 (0.23)
	IPC	4.3 ± 0.4	5.3 ± 0.5	5.8 ± 0.6			

*Data are mean ± SEM. BT, best time; TT, total time; %DC, percent sprint performance decrement; CT, control; IPC, ischemic preconditioning; $\eta_{\text{p}}^{2}$, partial eta square; n = 15 for all variables*.

## Discussion

Our main finding was that IPC increased HRR60s which, in addition to being a valid (Kannankeril et al., 2004) and reproducible parameter (Al Haddad et al., 2011; Dupuy et al., 2012; Bonato et al., 2018), is the most used method to assess the post-exercise cardiac autonomic control in clinical and sports settings (Buchheit, 2014; Peçanha et al., 2014). This result is novel and may therefore have practical implications. However, contrary to our hypothesis, the IPC-mediated cardiac autonomic control improvement was not accompanied by changes in $\dot{\text{V}}{\text{O}}_{2}$, $\dot{\text{V}}{\text{O}}_{2}$, RER, and blood [Lac-]. Thus, collectively, the results indicate that IPC improved an important index of the short-term recovery of cardiac autonomic control from RSE, regardless of change in surrogates of energy metabolism responses to RSE.

### Effect of IPC on post-exercise cardiac autonomic control

In our study IPC did not change HRpeak. This finding is similar to those reported by studies that investigated the IPC effect on HRpeak during high-intensity intermittent exercise (Marocolo et al., 2017; Zinner et al., 2017) or HRpeak during ramp incremental exercise (de Groot et al., 2010; Crisafulli et al., 2011; Sabino-Carvalho et al., 2017). As far as we know, only two studies have reported the IPC effect on post-exercise cardiovascular parameters. Both studies used handgrip exercise and employed circulatory occlusion during recovery from exercise to isolate the activation of the muscle metaboreflex. Incognito et al. (2017) reported IPC did not change arterial pressure, HR and muscle sympathetic nerve activity during exercise and post-exercise circulatory occlusion. Mulliri et al. (2016) reported IPC did not change central and peripheral hemodynamic parameters during exercise, but IPC decreased mean arterial pressure during post-exercise circulatory occlusion due to a venous return-induced reduction of stroke volume and cardiac output. Of note, however, Incognito et al. (2017) and Mulliri et al. (2016) did not provide enough data to interpret the IPC effect under normal free flow recovery from exercise, like we did in the present study. In addition, cardiovascular responses to handgrip exercise are very different from those provoked by dynamic exercise involving large muscle mass (Lewis et al., 1985). For example, Incognito et al. (2017) and Mulliri et al. (2016) reported mean HR to achieve 102 and 72 bpm, respectively. In contrast, in our study HR surpassed 180 bpm. Therefore, methodological dissimilarities preclude comparison of the IPC effect on post-exercise cardiovascular parameters between former studies (Mulliri et al., 2016; Incognito et al., 2017) and the present one.

We found that IPC did not change HRR up to 25 s, T30, HRR Tau and RMSSD. Conversely, IPC consistently lowered HR from 30 to 60 s, leading to increased HRR60s after all sets. Thus, our results indicate that IPC only had an effect on the second half of the short-term HRR. A possible explanation for the divergent IPC effect on short-term HRR indexes is that the RSE deleterious effect on HRR was so powerful that HR did not decay within approximately the first 10 s of recovery (i.e., onset HRR). As a result, the T30 index which relies on onset recovery data may have not been sensitive to assess the short-term component of post-RSE HRR. This phenomenon may dependent on the exercise intensity, given that it has also been reported by other studies that assessed post-RSE HRR (Buchheit et al., 2007; Nakamura et al., 2009; Del Rosso et al., 2017), but not by studies that assessed HRR after submaximal or maximal incremental exercise in healthy subjects (Imai et al., 1994; Kannankeril et al., 2004). Another point is that the IPC effect in the present study may have not been large enough to overcome the variability of parameters with low-to-moderate reproducibility, such as T30 and RMSSD. On the other hand, the absence of IPC effect on the HRR Tau was unequivocal, due to equal mean values between IPC and CT, high Student's *t*-test *P*-value and reasonable reproducibility of this parameter (Bonato et al., 2018). These facts therefore clearly support that IPC did not change long-term post-RSE HRR.

Previous studies have used systemic pharmacological blockade of vagal (i.e., muscarinic) and sympathetic (i.e., beta) receptors aiming to dissect the contribution of each branch of the autonomic nervous system to HRR (Imai et al., 1994; Kannankeril et al., 2004; Goldberger et al., 2006). These studies showed both short- and long-term HRR are largely determined by cardiac vagal reactivation post-submaximal exercise, with a negligible influence of the sympathetic branch. However, no study has investigated the autonomic contribution to HRR after supramaximal exercise (i.e., exercise performed at velocity or workload higher than peak velocity or workload of an incremental exercise test), such as RSE, which may not be the same as post-maximal exercise. Firstly, because our study and others showed RMSSD almost did not recover post-RSE (Buchheit et al., 2007; Nakamura et al., 2009; Del Rosso et al., 2017), which is not the case post-maximal exercise (Goldberger et al., 2006). Secondly, because the level of circulating catecholamines follows exercise-induced lactic acidosis (Mazzeo and Marshall, 1989) and RSE usually leads to greater lactic acidosis than maximal exercise (Buchheit et al., 2007; Sabino-Carvalho et al., 2017). Thirdly, because intense muscle metaboreflex activation during recovery from exercise under circulatory occlusion increases cardiac sympathoexcitation and offsets the influence of cardiac vagal activation on HR (Fisher et al., 2010). In sum, although cardiac vagal reactivation possibly plays a crucial role for short-term post-RSE HRR, sympathoexcitation is greater during RSE than maximal exercise. Consequently, cardiac sympathetic activity perhaps restrains to some extent short-term post-RSE HRR, and cardiac sympathetic withdrawal may importantly contribute to long-term post-RSE HRR. Thus, in our study, IPC-induced acceleration of HRR60s was possibly mediated by greater cardiac vagal reactivation, but lower cardiac sympathetic restraint cannot be disregarded. In contrast, as IPC did not change HRR after 60 s of recovery and RMSSD along 360 s of recovery, cardiac vagal reactivation and, particularly, cardiac sympathetic withdrawal likely did not change during the long-term component of cardiac autonomic control recovery.

### Effect of IPC on energy metabolism responses

IPC increased peak pulmonary oxygen uptake ($\dot{\text{V}}{\text{O}}_{2}$) in a sample mostly composed by men (de Groot et al., 2010; Cruz et al., 2015) and decreased blood lactate concentration [Lac-] in men (Bailey et al., 2012) during incremental dynamic exercise. In contrast, Patterson et al. (2015) showed no effect of IPC on $\dot{\text{V}}{\text{O}}_{2}$, $\dot{\text{V}}{\text{O}}_{2}$, and blood [Lac-] during twelve 6-s cycling sprints in men. Gibson et al. (2015) reported IPC to reduce blood [Lac-] in women, but not men, post five 6-s cycling sprints. More recently, Griffin et al. (2018) did not find an effect of IPC on blood [Lac-] during three sets of six shuttle run sprints in men. The reason for the dissimilar IPC effect on surrogates of energy metabolism during incremental dynamic exercise vs. RSE is still unclear. One possibility could be that the IPC dose has been insufficient for RSE. Thus, herein we employed a repeated IPC protocol. Nevertheless, we also found that IPC changed none of the assessed surrogates of energy metabolism. This result consequently suggests that the IPC dose may not be an issue. Possible modulators of the IPC effect, such as gender (Gibson et al., 2015), physical fitness (Sabino-Carvalho et al., 2017), and time between IPC application and exercise assessments (Lisbôa et al., 2017) should then be taken into consideration by next studies. Still of note, our study and others (de Groot et al., 2010; Crisafulli et al., 2011; Bailey et al., 2012; Cruz et al., 2015; Gibson et al., 2015; Patterson et al., 2015; Griffin et al., 2018) have measured $\dot{\text{V}}{\text{O}}_{2}$ and $\dot{\text{V}}{\text{O}}_{2}$ via analysis of breathing air and [Lac-] via analysis of capillary blood, but these methods only provide indirect information with regards to muscle aerobic and anaerobic responses. Thus, other methods should be employed in the future to confirm the IPC effect on energy metabolism responses to RSE.

As IPC did not change energy metabolism surrogates during a RSE task, the acceleration of HRR60s was possibly mediated by other factors than energy metabolism responses to RSE. In this sense, IPC has been linked with release of humoral factors by preconditioned tissues (e.g., adenosine, bradykinin, and calcitonin), as well as by activation of afferent neural pathways in preconditioned tissues (e.g., C- and Aδ-fibers) (Gourine and Gourine, 2014). Humoral and neural mechanisms have thus been considered triggers of IPC-induced vagal activation during ischemia-reperfusion injury protocols (Gourine and Gourine, 2014). Therefore, direct vagal activation via neural and humoral mechanisms likely underlies the IPC effect on the HRR60s, rather than an indirect IPC effect on energy metabolism responses to exercise.

Some studies have shown that IPC can enhance exercise performance due to a placebo effect rather than a specific IPC effect (Marocolo et al., 2015, 2016; Sabino-Carvalho et al., 2017). Noteworthy, the IPC placebo effect on exercise performance was not accompanied by change in energy metabolism and HR responses to exercise (Marocolo et al., 2015, 2016; Sabino-Carvalho et al., 2017). Our experimental design did not allow dissecting specific IPC effects from placebo effects. However, based on the aforementioned studies (Clark et al., 2000; Foad et al., 2008; Marocolo et al., 2015, 2016; Sabino-Carvalho et al., 2017), it is possible that sprints time were more prone to placebo and nocebo effects than physiological measurements.

### Implications

HRR60s percent change (12.8% for IPC main effect) surpassed the smallest worthwhile change (6%, calculated as 0.2 × between-subjects standard deviation for all sets in the CT condition) and the coefficient of variation elsewhere reported (i.e., 11%) (Bonato et al., 2018). In addition, the HRR60s absolute change in the present study (i.e., 4 bpm) was somewhat comparable to the absolute change provoked by 8 weeks of high-intensity interval training in patients with coronary disease (i.e., 6 bpm) (Villelabeitia-Jaureguizar et al., 2017) and by 3 weeks of high-intensity interval training in well-trained cyclists (i.e., 5 bpm) (Lamberts et al., 2009). The IPC effect herein observed therefore achieved a magnitude that could carry practical implications for clinical populations and athletes which deserves investigation by next studies. With regard to clinical populations, studies in dogs strongly support that the higher the HRR and the cardiac vagal outflow to the heart, the lower is the chance of developing ventricular fibrillation and dyeing after induction of acute myocardial ischemia during exercise (Vanoli et al., 1991; Smith et al., 2005). Although we assessed healthy young men, our results suggest IPC could carry cardioprotective benefits for subjects with cardiovascular risk factors or stablished cardiovascular diseases that engage in RSE training or practice of sports that may involve RSE, such as soccer, basketball, tennis, etc. With regard to athletes, a better post-exercise cardiac autonomic control may indicate greater readiness to train at high-intensity (Borresen and Lambert, 2007; Kiviniemi et al., 2010; Buchheit, 2014; Capostagno et al., 2014). Thus, the administration of IPC before high-intensity interval training sessions could enhance athletes' training tolerance, resulting in amplified or accelerated generation of chronic adaptations.

## Conclusion

IPC accelerated to some extent the short-term recovery, but did not change the long-term recovery of cardiac autonomic control from RSE, and such accelerator effect was not accompanied by any IPC effect on surrogates of energy metabolism responses to RSE.

## Datasets availability

The raw data supporting the conclusions of this manuscript will be made available by the authors, without undue reservation, to any qualified researcher.

## Author contributions

TL, JS, AS, and BS conception and design of the study. TL, JS-C, TF, and BS data acquisition. TL and BS data analysis and interpretation. TL and BS wrote the manuscript. TL, JS-C, TF, JS, AS, and BS critical review of the manuscript. All authors contributed to manuscript revision, read and approved the submitted version.

### Conflict of interest statement

The authors declare that the research was conducted in the absence of any commercial or financial relationships that could be construed as a potential conflict of interest.

## References

[B1] Al Haddad H. Laursen P. B. Chollet D. Ahmaidi S. Buchheit M. (2011). Reliability of resting and postexercise heart rate measures. Int. J. Sports Med. 32, 598–605. 10.1055/s-0031-1275356 21574126

[B2] Bailey T. G. Jones H. Gregson W. Atkinson G. Cable N. T. Thijssen D. H. (2012). Effect of ischemic preconditioning on lactate accumulation and running performance. Med. Sci. Sports Exerc. 44, 2084–2089. 10.1249/MSS.0b013e318262cb17 22843115

[B3] Barbosa T. C. Machado A. C. Braz I. D. Fernandes I. A. Vianna L. C. Nobrega A. C.. (2015). Remote ischemic preconditioning delays fatigue development during handgrip exercise. Scand. J. Med. Sci. Sports 25, 356–364. 10.1111/sms.12229 24731023

[B4] Basalay M. Barsukevich V. Mastitskaya S. Mrochek A. Pernow J. Sjoquist P. O.. (2012). Remote ischaemic pre- and delayed postconditioning - similar degree of cardioprotection but distinct mechanisms. Exp. Physiol. 97, 908–917. 10.1113/expphysiol.2012.064923 22427438 PMC3470925

[B5] Bonato M. Meloni A. Merati G. La Torre A. Agnello L. Vernillo G. (2018). Effect of repeated-sprints on the reliability of short-term parasympathetic reactivation. PLoS ONE 13:e0192231. 10.1371/journal.pone.0192231 29408911 PMC5800600

[B6] Borresen J. Lambert M. I. (2007). Changes in heart rate recovery in response to acute changes in training load. Eur. J. Appl. Physiol. 101, 503–511. 10.1007/s00421-007-0516-6 17687564

[B7] Buchheit M. (2014). Monitoring training status with HR measures: do all roads lead to Rome? Front. Physiol. 5:73. 10.3389/fphys.2014.00073 24578692 PMC3936188

[B8] Buchheit M. Laursen P. B. Ahmaidi S. (2007). Parasympathetic reactivation after repeated sprint exercise. Am. J. Physiol. Heart Circ. Physiol. 293, H133–H141. 10.1152/ajpheart.00062.2007 17337589

[B9] Cabrera J. A. Ziemba E. A. Colbert R. Anderson L. B. Sluiter W. Duncker D. J.. (2012). Altered expression of mitochondrial electron transport chain proteins and improved myocardial energetic state during late ischemic preconditioning. Am. J. Physiol. Heart Circ. Physiol. 302, H1974–H1982. 10.1152/ajpheart.00372.2011 22389388 PMC3362109

[B10] Capostagno B. Lambert M. I. Lamberts R. P. (2014). Standardized versus customized high-intensity training: effects on cycling performance. Int. J. Sports Physiol. Perform. 9, 292–301. 10.1123/ijspp.2012-0389 23881116

[B11] Clark V. R. Hopkins W. G. Hawley J. A. Burke L. M. (2000). Placebo effect of carbohydrate feedings during a 40-km cycling time trial. Med. Sci. Sports Exerc. 32, 1642–1647. 10.1097/00005768-200009000-00019 10994918

[B12] Cocking S. Cable N. T. Wilson M. G. Green D. J. Thijssen D. H. J. Jones H. (2018). Conduit artery diameter during exercise is enhanced after local, but not remote, ischemic preconditioning. Front. Physiol. 9:435 10.3389/fphys.2018.00435 29740345 PMC5928322

[B13] Cohen J. (1988). Statistical Power Analysis for the Behavioral Sciences. New York, NY: Lawrence Erlbaum Associates.

[B14] Cole C. R. Blackstone E. H. Pashkow F. J. Snader C. E. Lauer M. S. (1999). Heart-rate recovery immediately after exercise as a predictor of mortality. N. Engl. J. Med. 341, 1351–1357. 10.1056/NEJM199910283411804 10536127

[B15] Coote J. H. (2010). Recovery of heart rate following intense dynamic exercise. Exp. Physiol. 95, 431–440. 10.1113/expphysiol.2009.047548 19837772

[B16] Crisafulli A. Tangianu F. Tocco F. Concu A. Mameli O. Mulliri G. (2011). Ischemic preconditioning of the muscle improves maximal exercise performance but not maximal oxygen uptake in humans. J. Appl. Physiol. 111, 530–536. 10.1152/japplphysiol.00266.2011 21617078

[B17] Cruz R. S. De Aguiar R. A. Turnes T. Pereira K. L. Caputo F. (2015). Effects of ischemic preconditioning on maximal constant-load cycling performance. J. Appl. Physiol. 119, 961–967. 10.1152/japplphysiol.00498.2015 26359484

[B18] de Groot P. C. Thijssen D. H. Sanchez M. Ellenkamp R. Hopman M. T. (2010). Ischemic preconditioning improves maximal performance in humans. Eur. J. Appl. Physiol. 108, 141–146. 10.1007/s00421-009-1195-2 19760432 PMC2793394

[B19] Del Rosso S. Nakamura F. Y. Boullosa D. A. (2017). Heart rate recovery after aerobic and anaerobic tests: is there an influence of anaerobic speed reserve? J. Sports Sci. 35, 820–827. 10.1080/02640414.2016.1166391 27018761

[B20] Dupuy O. Mekary S. Berryman N. Bherer L. Audiffren M. Bosquet L. (2012). Reliability of heart rate measures used to assess post-exercise parasympathetic reactivation. Clin. Physiol. Funct. Imaging 32, 296–304. 10.1111/j.1475-097X.2012.01125.x 22681607

[B21] Enko K. Nakamura K. Yunoki K. Miyoshi T. Akagi S. Yoshida M.. (2011). Intermittent arm ischemia induces vasodilatation of the contralateral upper limb. J. Physiol. Sci. 61, 507–513. 10.1007/s12576-011-0172-9 21901641 PMC10718035

[B22] Ferreira T. N. Sabino-Carvalho J. L. Lopes T. R. Ribeiro I. C. Succi J. E. DA Silva A. C.. (2016). Ischemic preconditioning and repeated sprint swimming: a placebo and nocebo study. Med. Sci. Sports Exerc. 48, 1967–1975. 10.1249/MSS.0000000000000977 27187105

[B23] Fisher J. P. Seifert T. Hartwich D. Young C. N. Secher N. H. Fadel P. J. (2010). Autonomic control of heart rate by metabolically sensitive skeletal muscle afferents in humans. J. Physiol. 588, 1117–1127. 10.1113/jphysiol.2009.185470 20142272 PMC2852999

[B24] Fitzsimons M. Dawson B. Ward D. Wilkinson A. (1993). Cycling and running tests of repeated sprint ability. Aust. J. Sci. Med. Sport 25, 82–87.

[B25] Foad A. J. Beedie C. J. Coleman D. A. (2008). Pharmacological and psychological effects of caffeine ingestion in 40-km cycling performance. Med. Sci. Sports Exerc. 40, 158–165. 10.1249/mss.0b013e3181593e02 18091009

[B26] Gamelin F. X. Berthoin S. Bosquet L. (2006). Validity of the polar S810 heart rate monitor to measure R-R intervals at rest. Med. Sci. Sports Exerc. 38, 887–893. 10.1249/01.mss.0000218135.79476.9c 16672842

[B27] Garlid K. D. Dos Santos P. Xie Z. J. Costa A. D. Paucek P. (2003). Mitochondrial potassium transport: the role of the mitochondrial ATP-sensitive K(+) channel in cardiac function and cardioprotection. Biochim. Biophys. Acta 30, 1–21. 10.1016/S0005-2728(03)00109-9 14507424

[B28] Gibson N. Mahony B. Tracey C. Fawkner S. Murray A. (2015). Effect of ischemic preconditioning on repeated sprint ability in team sport athletes. J. Sports Sci. 33, 1182–1188. 10.1080/02640414.2014.988741 25517761

[B29] Gist N. H. Fedewa M. V. Dishman R. K. Cureton K. J. (2014). Sprint interval training effects on aerobic capacity: a systematic review and meta-analysis. Sports Med. 44, 269–279. 10.1007/s40279-013-0115-0 24129784

[B30] Goldberger J. J. Le F. K. Lahiri M. Kannankeril P. J. Ng J. Kadish A. H. (2006). Assessment of parasympathetic reactivation after exercise. Am. J. Physiol. Heart Circ. Physiol. 290, H2446–H2452. 10.1152/ajpheart.01118.2005 16415073

[B31] Gourine A. Gourine A. V. (2014). Neural mechanisms of cardioprotection. Physiology 29, 133–140. 10.1152/physiol.00037.2013 24583769 PMC3949205

[B32] Griffin P. J. Hughes L. Gissane C. Patterson S. D. (2018). Effects of local versus remote ischemic preconditioning on repeated sprint running performance. J. Sports Med. Phys. Fitness 2, 08400–08401. 10.23736/S0022-4707.18.08400-1 29722251

[B33] Hausenloy D. J. Yellon D. M. (2010). The second window of preconditioning (SWOP) where are we now? Cardiovasc. Drugs. Ther. 24, 235–254. 10.1007/s10557-010-6237-9 20496105

[B34] Hausswirth C. Bigard A. X. Le Chevalier J. M. (1997). The Cosmed K4 telemetry system as an accurate device for oxygen uptake measurements during exercise. Int. J. Sports Med. 18, 449–453. 10.1055/s-2007-972662 9351691

[B35] Imai K. Sato H. Hori M. Kusuoka H. Ozaki H. Yokoyama H.. (1994). Vagally mediated heart rate recovery after exercise is accelerated in athletes but blunted in patients with chronic heart failure. J. Am. Coll. Cardiol. 24, 1529–1535. 10.1016/0735-1097(94)90150-3 7930286

[B36] Impellizzeri F. M. Rampinini E. Castagna C. Bishop D. Ferrari Bravo D. Tibaudi A.. (2008). Validity of a repeated-sprint test for football. Int. J. Sports Med. 29, 899–905. 10.1055/s-2008-1038491 18415931

[B37] Incognito A. V. Doherty C. J. Lee J. B. Burns M. J. Millar P. J. (2017). Ischemic preconditioning does not alter muscle sympathetic responses to static handgrip and metaboreflex activation in young healthy men. Physiol. Rep. 5:13342 10.14814/phy2.13342 PMC5532483 28720715

[B38] Jeffries O. Waldron M. Pattison J. R. Patterson S. D. (2018). Enhanced local skeletal muscle oxidative capacity and microvascular blood flow following 7-day ischemic preconditioning in healthy humans. Front. Physiol. 9:463. 10.3389/fphys.2018.00463 29867526 PMC5954802

[B39] Jones H. Hopkins N. Bailey T. G. Green D. J. Cable N. T. Thijssen D. H. (2014). Seven-day remote ischemic preconditioning improves local and systemic endothelial function and microcirculation in healthy humans. Am. J. Hypertens. 27, 918–925. 10.1093/ajh/hpu004 24627443

[B40] Kannankeril P. J. Le F. K. Kadish A. H. Goldberger J. J. (2004). Parasympathetic effects on heart rate recovery after exercise. J. Investig. Med. 52, 394–401. 10.1136/jim-52-06-34 15612453

[B41] Kharbanda R. K. Mortensen U. M. White P. A. Kristiansen S. B. Schmidt M. R. Hoschtitzky J. A.. (2002). Transient limb ischemia induces remote ischemic preconditioning *in vivo*. Circulation 106, 2881–2883. 10.1161/01.CIR.0000043806.51912.9B 12460865

[B42] Kiviniemi A. M. Hautala A. J. Kinnunen H. Nissila J. Virtanen P. Karjalainen J.. (2010). Daily exercise prescription on the basis of HR variability among men and women. Med. Sci. Sports Exerc. 42, 1355–1363. 10.1249/MSS.0b013e3181cd5f39 20575165

[B43] Lamberts R. P. Swart J. Noakes T. D. Lambert M. I. (2009). Changes in heart rate recovery after high-intensity training in well-trained cyclists. Eur. J. Appl. Physiol. 105, 705–713. 10.1007/s00421-008-0952-y 19101720

[B44] Lewis S. F. Snell P. G. Taylor W. F. Hamra M. Graham R. M. Pettinger W. A.. (1985). Role of muscle mass and mode of contraction in circulatory responses to exercise. J. Appl. Physiol. 58, 146–151. 10.1152/jappl.1985.58.1.146 3968005

[B45] Lisbôa F. D. Turnes T. Cruz R. S. Raimundo J. A. Pereira G. S. Caputo F. (2017). The time dependence of the effect of ischemic preconditioning on successive sprint swimming performance. J. Sci. Med. Sport 20, 507–511. 10.1016/j.jsams.2016.09.008 27717653

[B46] Loukogeorgakis S. P. Panagiotidou A. T. Broadhead M. W. Donald A. Deanfield J. E. Macallister R. J. (2005). Remote ischemic preconditioning provides early and late protection against endothelial ischemia-reperfusion injury in humans: role of the autonomic nervous system. J. Am. Coll. Cardiol. 46, 450–456. 10.1016/j.jacc.2005.04.044 16053957

[B47] Marocolo I. C. Da Mota G. R. Londe A. M. Patterson S. D. Barbosa Neto O. Marocolo M. (2017). Acute ischemic preconditioning does not influence high-intensity intermittent exercise performance. Peer J. 30:e4118 10.7717/peerj.4118 PMC5712465 29204325

[B48] Marocolo M. da Mota G. R. Pelegrini V. Appell Coriolano H. J. (2015). Are the beneficial effects of ischemic preconditioning on performance partly a placebo effect? Int. J. Sports Med. 36, 822–825. 10.1055/s-0035-1549857 26058479

[B49] Marocolo M. Willardson J. M. Marocolo I. C. da Mota G. R. Simao R. Maior A. S. (2016). Ischemic preconditioning and placebo intervention improves resistance exercise performance. J. Strength Cond. Res. 30, 1462–1469. 10.1519/JSC.0000000000001232 26466134

[B50] Mastitskaya S. Marina N. Gourine A. Gilbey M. P. Spyer K. M. Teschemacher A. G.. (2012). Cardioprotection evoked by remote ischaemic preconditioning is critically dependent on the activity of vagal pre-ganglionic neurones. Cardiovasc. Res. 95, 487–494. 10.1093/cvr/cvs212 22739118 PMC3422080

[B51] Mazzeo R. S. Marshall P. (1989). Influence of plasma catecholamines on the lactate threshold during graded exercise. J. Appl. Physiol. 67, 1319–1322. 10.1152/jappl.1989.67.4.1319 2793730

[B52] Mulliri G. Sainas G. Magnani S. Palazzolo G. Milia N. Orrù A.. (2016). Ischemic preconditioning reduces hemodynamic response during metaboreflex activation. Am. J. Physiol. Regul. Integr. Comp. Physiol. 310, R777–R787. 10.1152/ajpregu.00429.2015 26936782 PMC5000777

[B53] Nakamura F. Y. Soares-Caldeira L. F. Laursen P. B. Polito M. D. Leme L. C. Buchheit M. (2009). Cardiac autonomic responses to repeated shuttle sprints. Int. J. Sports Med. 30, 808–813. 10.1055/s-0029-1234055 19685413

[B54] Pang C. Y. Yang R. Z. Zhong A. Xu N. Boyd B. Forrest C. R. (1995). Acute ischaemic preconditioning protects against skeletal muscle infarction in the pig. Cardiovasc. Res. 29, 782–788. 10.1016/S0008-6363(96)88613-5 7656281

[B55] Patterson S. D. Bezodis N. E. Glaister M. Pattison J. R. (2015). The effect of ischemic preconditioning on repeated sprint cycling performance. Med. Sci. Sports Exerc. 47, 1652–1658. 10.1249/MSS.0000000000000576 25412297

[B56] Peçanha T. Silva-Junior N. D. Forjaz C. L. (2014). Heart rate recovery: autonomic determinants, methods of assessment and association with mortality and cardiovascular diseases. Clin. Physiol. Funct. Imaging 34, 327–339. 10.1111/cpf.12102 24237859

[B57] Piiper J. Spiller P. (1970). Repayment of O2 debt and resynthesis of high-energy phosphates in gastrocnemius muscle of the dog. J. Appl. Physiol. 28, 657–662. 10.1152/jappl.1970.28.5.657 5442264

[B58] Poole D. C. Jones A. M. (2012). Compr. Physiol. 2, 933–996. 10.1002/cphy.c100072 23798293

[B59] Rampinini E. Bishop D. Marcora S. M. Ferrari Bravo D. Sassi R. Impellizzeri F. M. (2007). Validity of simple field tests as indicators of match-related physical performance in top-level professional soccer players. Int. J. Sports Med. 28, 228–235. 10.1055/s-2006-924340 17024621

[B60] Rossiter H. B. Ward S. A. Kowalchuk J. M. Howe F. A. Griffiths J. R. Whipp B. J. (2002). Dynamic asymmetry of phosphocreatine concentration and O(2) uptake between the on- and off-transients of moderate- and high-intensity exercise in humans. J. Physiol. 541, 991–1002. 10.1113/jphysiol.2001.012910 12068057 PMC2290368

[B61] Sabino-Carvalho J. L. Lopes T. R. Obeid-Freitas T. Ferreira T. N. Succi J. E. Silva A. C. (2017). Effect of ischemic preconditioning on endurance performance does not surpass placebo. Med. Sci. Sports Exerc. 49, 124–132. 10.1249/MSS.0000000000001088 27580156

[B62] Serpiello F. R. Mckenna M. J. Stepto N. K. Bishop D. J. Aughey R. J. (2011). Performance and physiological responses to repeated-sprint exercise: a novel multiple-set approach. Eur. J. Appl. Physiol. 111, 669–678. 10.1007/s00421-010-1687-0 20957389

[B63] Smith L. L. Kukielka M. Billman G. E. (2005). Heart rate recovery after exercise: a predictor of ventricular fibrillation susceptibility after myocardial infarction. Am. J. Physiol. Heart Circ. Physiol. 288:24. 10.1152/ajpheart.00785.2004 15563535

[B64] Stork M. J. Banfield L. E. Gibala M. J. Martin Ginis K. A. (2017). A scoping review of the psychological responses to interval exercise: is interval exercise a viable alternative to traditional exercise? Health. Psychol. Rev. 11, 324–344. 10.1080/17437199.2017.1326011 28460601

[B65] Task Force (1996). Heart rate variability: standards of measurement, physiological interpretation and clinical use. Circulation 93, 1043–1065. 10.1161/01.CIR.93.5.1043 8598068

[B66] Thaveau F. Zoll J. Rouyer O. Chafke N. Kretz J. G. Piquard F.. (2007). Ischemic preconditioning specifically restores complexes I and II activities of the mitochondrial respiratory chain in ischemic skeletal muscle. J. Vasc. Surg. 46, 541–547. 10.1016/j.jvs.2007.04.075 17826242

[B67] Vanderlei L. C. Silva R. A. Pastre C. M. Azevedo F. M. Godoy M. F. (2008). Comparison of the Polar S810i monitor and the ECG for the analysis of heart rate variability in the time and frequency domains. Braz. J. Med. Biol. Res. 41, 854–859. 10.1590/S0100-879X2008005000039 18853042

[B68] Vanoli E. De Ferrari G. M. Stramba-Badiale M. Hull S. S. Jr. Foreman R. D. Schwartz P. J. (1991). Vagal stimulation and prevention of sudden death in conscious dogs with a healed myocardial infarction. Circ. Res. 68, 1471–1481. 10.1161/01.RES.68.5.1471 2019002

[B69] Villelabeitia-Jaureguizar K. Vicente-Campos D. Senen A. B. Jimenez V. H. Garrido-Lestache M. E. B. Chicharro J. L. (2017). Effects of high-intensity interval versus continuous exercise training on post-exercise heart rate recovery in coronary heart-disease patients. Int. J. Cardiol. 244, 17–23. 10.1016/j.ijcard.2017.06.067 28648356

[B70] Weippert M. Kumar M. Kreuzfeld S. Arndt D. Rieger A. Stoll R. (2010). Comparison of three mobile devices for measuring R-R intervals and heart rate variability: polar S810i, Suunto t6 and an ambulatory ECG system. Eur. J. Appl. Physiol. 109, 779–786. 10.1007/s00421-010-1415-9 20225081

[B71] Zinner C. Born D. P. Sperlich B. (2017). Ischemic preconditioning does not alter performance in multidirectional high-intensity intermittent exercise. Front. Physiol. 8:1029 10.3389/fphys.2017.01029 29311963 PMC5732929

